# Addition of Sodium Pyruvate to Stored Red Blood Cells Attenuates Liver Injury in a Murine Transfusion Model

**DOI:** 10.1155/2016/3549207

**Published:** 2016-09-26

**Authors:** Sha Xia, Gan Chen, Bo Wang, Yujing Yin, Zhenwei Sun, Jingxiang Zhao, Penglong Li, Lian Zhao, Hong Zhou

**Affiliations:** Beijing Institute of Transfusion Medicine, Beijing Key Laboratory of Blood Safety and Supply Technologies, No. 27 Taiping Road, Haidian, Beijing, China

## Abstract

RBCs undergo numerous changes during storage and stored RBCs may induce adverse effects, ultimately resulting in organ injury in transfusion recipients. We tested the hypothesis that the addition of SP to stored RBCs would improve the quality of the stored RBCs and mitigate liver injury after transfusion in a murine model. RBCs were harvested from C57BL/6J mice and stored for 14 days in CPDA-1 containing either a solution of SP in saline or saline alone. Haemolysis, the 24-hour posttransfusion recovery, the oxygen-carrying capacity, and the SOD activity of stored RBCs were evaluated. The plasma biochemistry, hepatic MDA level, MPO activity, IL-6, TNF-*α* concentrations, and histopathology were measured two hours after the transfusion of stored RBCs. Compared with RBCs stored in CPDA-1 and saline, the addition of SP to stored RBCs restored their oxygen-carrying capacity and SOD activity, reduced the AST activity, BUN concentrations, and LDH activity in the plasma, and decreased the MDA level, MPO activity, and concentrations of IL-6 and TNF-*α* in the liver. These data indicate that the addition of SP to RBCs during storage has a beneficial effect on storage lesions* in vitro* and subsequently alleviates liver injury after the transfusion of stored RBCs* in vivo*.

## 1. Introduction

Red blood cell (RBC) transfusion is a lifesaving therapy for patients with haemorrhagic shock, anaemia, and surgery. To augment utilization and minimize waste, RBCs are often refrigerated and stored for several days in clinical situations. During storage, RBCs undergo a series of structural and functional alterations, referred to as the “storage lesion” [1–3]. Cellular ATP and 2,3-DPG depletion have been observed during storage, and these changes influence the survival of transfused RBCs and their oxygen-carrying capacity [4–6]. Additionally, the loss of cellular antioxidation capacity results in the generation of reactive oxygen species (ROS) during storage, which increases protein and lipid oxidation in stored RBCs [7]. This damage may directly stimulate the production of inflammation mediators and subsequently cause organ injury including liver injury in some recipient after the transfusion of stored RBCs [8, 9], which may be associated with increased morbidity and mortality [10–12].

The Food and Drug Administration (FDA) has approved the storage of RBCs in storage solutions. Specifically, RBCs can be stored for up to 42 days without exceeding 1% haemolysis and a minimum of 75% of transfused RBCs survival 24 h after transfusion [13–15]. Many studies have attempted to reduce storage lesions by improving the composition of the storage solution. The addition of nutrients and antioxidant was previously demonstrated to alleviate storage lesions during RBC storage. The addition of exogenous glucose can extend the storage of RBCs by decreasing haemolysis and supplying energy [16, 17]. Stowell et al. indicated that the addition of ascorbic acid solution benefited the recovery and immunogenicity of RBCs during storage [4]. This study also indicated that simple antioxidants could not decrease inflammation in a murine transfusion model. Herein, we hypothesized that a multifunctional additive solution can provide a substrate for energy metabolism and exert an antioxidant effect during RBC storage which can reduce storage lesions as well as decrease oxidative stress and inflammation after transfusion in a murine transfusion model.

Pyruvate is a three-carbon molecule that is endogenously produced during glycolysis. Pyruvate can be converted into ATP in the tricarboxylic acid (TCA) cycle and has also been demonstrated to act as an antioxidant [18]. Sodium pyruvate (SP) was reported to attenuate tissue damage in haemorrhagic shock, brain damage, and ischemia-reperfusion injury models [19–22]. In addition, previous studies indicated that pyruvate can maintain ATP and 2,3-DPG levels during RBC storage* in vitro* [23, 24], and these results suggested that the addition of SP during RBC storage might alleviate storage lesions, which need to be further studied. Moreover, the effects of adding SP during RBC storage on organ injury after the transfusion of stored RBCs* in vivo* are unknown. We hypothesized that the addition of SP during RBC storage can reduce storage lesions and subsequently decrease organ injury in recipients after transfusion.

The murine storage RBC model is widely used to study the damage to RBCs during storage [9, 25]. Previous studies showed that murine RBCs stored for two weeks were similar to human RBCs stored for six weeks and underwent similar physiological and biochemical changes during storage [7, 26–28]. The effects of SP on the transfusion of stored RBCs were evaluated in a murine model. We found that SP can restore the oxygen-carrying capacity of RBCs and decrease oxidative stress and inflammation in the liver after the transfusion of stored RBCs in a murine model.

## 2. Materials and Methods

### 2.1. Animals

The study was approved by the Institutional Animal Care and Use Committee of the Academy of Military Medical Sciences (IACUC number AMMS-2014-027). All efforts were made to minimize the number of animals used and their suffering. Eight- to ten-week-old wild-type male C57BL/6J mice were purchased from Vital River (Beijing, China) and used after an acclimation period of at least five to seven days at 25°C in a 12 h light/12 h dark cycle.

### 2.2. SP Stock Preparation and Addition

SP was purchased (Sigma, St. Louis, MO, USA), and stock solutions of SP were prepared in saline solution at final concentrations of 325 mmol/L and filtered through a 0.22 *μ*m vacuum filter. The SP stock solutions were then added to mouse RBCs at a 1 : 130 (vol/vol) ratio, and the final concentration of SP in the storage solutions was 2.5 mmol/L prior to centrifugation.

### 2.3. Mouse RBCs: Collection, Storage, and Transfusion

The mice were anesthetized with intraperitoneal injections of sodium pentobarbital (75 mg/kg) and mechanically ventilated at a respiratory rate of 120/min and tidal volume of 10 *μ*L/g using a Minivent mouse ventilator (Harvard Apparatus, Hugstetten, Germany). The body temperature was maintained at 36.5 ± 0.5°C using a heating pad (SOFTRON, TMS-201, Beijing, China). During the blood collection, surgical site was disinfected with 75% ethanol, and the mice were aseptically bled by cardiac puncture at sterile work tables, and the blood was collected in CPDA-1 (Sigma, St. Louis, MO, USA). The final CPDA-1 concentration used for storage was 14%. The whole blood collected from 20 to 30 mice was pooled, leukoreduced using a sterile high-efficiency leukoreduction filter (ZhiXing Bio S&T Co., Bengbu, China), centrifuged at 400 ×g for 15 min, and volume-reduced to a final hematocrit of 70% to 75% (Hb of 17 to 18 g/dL).

The RBCs were stored in two different solutions: (1) control group, RBCs stored in CPDA-1 plus saline, and (2) SP group, RBCs stored in CPDA-1 plus 2.5 mM SP. The RBCs were placed in 0.6 mL tubes (Corning, CA, USA) and stored at 4°C for up to 14 days.

Before transfusion, the stored RBCs were washed three times using 10 volumes of PBS and centrifuged at 400 ×g. After the final wash, the washed and stored RBCs were resuspended in PBS to a final Hb concentration similar to a final hematocrit of 70% to 75%. All recipient mice received 20% by volume of the total blood volume of the mouse via the tail vein. Two hours later, the mice were aseptically bled by cardiac puncture to assess the plasma biochemistry. The mice were then euthanized to obtain tissue samples. The tissue samples were washed with cold saline, snap-frozen in liquid nitrogen, and stored in liquid nitrogen until assayed.

### 2.4. Measurement and Calculations of* In Vitro* RBC Haemolysis

The blood gas was measured with an ABL90 FLEX Blood Gas Analyser (Radiometer, Copenhagen, Denmark) after 14 days of storage. The supernatant and total Hb, which were used to calculate the percentage of haemolysis, were measured using a free haemoglobin assay kit (Jiancheng Bioengineering Institute, Nanjing, China). The haemolysis rate was subsequently calculated as follows [29]:

(1) Haemolysis  rate%=1−Hct×supernatant  Hb g/dLTotal  Hb g/dL×100%.

### 2.5. Flow Cytometry Assessment of RBC Survival

RBCs were labelled with fluorescein isothiocyanate (FITC) (Sigma, St. Louis, MO, USA) as previously reported [30, 31]. A fluorescent dye solution was prepared by dissolving 100 mg of fluorescein isothiocyanate (FITC) in 10 mL of PBS. This solution was added to the diluent stored blood, for a final FITC concentration of 66 *μ*g/mL. Cells were incubated in this staining solution for 20 min at 37°C in the dark, and the labelled RBCs were washed three times in PBS and transfused into each recipient mouse (five mice per experimental group) via the tail vein. Ten minutes and 24 h after the transfusion, the recipients were retroorbitally bled into heparinized tubes, and 5 *μ*L of blood was diluted in 1 mL of FACS buffer. The percentage of fluorescent RBCs was measured by flow cytometry (BD FACSCalibur, MA, USA) using the percentage of fluorescent RBCs measured 10 min after transfusion as the reference value. The 24 h survival was determined based on the ratio of fluorescent RBCs at 24 h to the reference value.

### 2.6. Haemoglobin Oxygen Saturation Experiments

Blood oxygen saturation was measured with a Hemox Analyser (TCS Scientific Corporation, New Hope, PA, USA). Briefly, a suspension of erythrocytes containing 6 mg of haemoglobin was initially incubated at 37°C in 4 mL of Hemox buffer (30 mM TES (N-Tris hydroxymethyl-methyl-2-aminoethanesulfonic acid), 135 mM NaCl and 5 mM KCl, pH 7.40 ± 0.02 at 37°C; osmolarity 295 ± 10 mOsm/kg) and 0.1% BSA in the Hemox Analyser. After the addition of 10 *μ*L of antifoaming agent (TCS Scientific, New Hope, PA, USA), the pO_2_ and temperature were allowed to stabilize before measuring the oxygen saturation. The *P*_50_ value was then extrapolated on the *x*-axis as the point at which the O_2_ saturation was 50%.

### 2.7. Measurements of Plasma Biochemistry and Lactate Levels

The plasma aspartate aminotransferase (AST) activity, blood urea nitrogen (BUN) concentration, and lactate dehydrogenase (LDH) activity were evaluated using a Hitachi 7180 autoanalyser (Hitachi High-Technologies Corp., Tokyo, Japan). The lactate concentration was determined by lactic acid assay kit (Jiancheng Biological Institute, Nanjing, China) according to the manufacturer's recommendations.

### 2.8. Measurement of MDA Level, MPO Activity, and SOD Activity

The activity of SOD in stored RBCs was measured using a SOD Assay Kit (Jiancheng Biological Institute, Nanjing, China). Liver tissue was homogenized in cold normal saline to prepare a 10% homogenate and then centrifuged at 1,000 rpm and at 4°C for 6 min. The supernatant was then collected to measure the MDA levels and MPO activity using MDA concentration and MPO activity assay kits (Jiancheng Biological Institute, Nanjing, China) according to the manufacturer's recommendations, as previously reported [32]. The total hepatic protein levels were measured using a BCA protein assay kit (Biomed Biology Corporation, Beijing, China).

### 2.9. Measurements of Inflammatory Cytokines Levels

Liver tissue was homogenized on ice in 0.9% saline containing a protease inhibitor cocktail (Roche, Mannheim, Germany). The homogenates were centrifuged at 1000 ×g for 6 min at 4°C, and the supernatants were assayed for inflammatory cytokines levels. The interleukin- (IL-) 6 and TNF-*α* levels in supernatant were determined using an enzyme-linked immunosorbent assay (ELISA) kit (PeproTech, Rocky Hill, NJ, USA) according to the manufacturer's instructions as reported [25]. The values are expressed as pg/mg protein.

### 2.10. Liver Histology

Two hours after transfusion, the livers were fixed with 4% paraformaldehyde and embedded in paraffin. The sections were stained with hematoxylin and eosin (H&E) to observe histological changes [33]. The histological changes in the liver were examined by light microscopy in a blinded fashion. The liver injury was assessed in the inflammatory cell infiltration and scored on a 4-point scale (0, none; 1, slight; 2, moderate; 3, severe) as previously described [34].

### 2.11. Statistical Analysis

The data are presented as the means ± standard deviations (SD). All analyses were conducted using statistical software (SAS Institute Inc., Cary, NC, USA). The sample numbers were chosen based on the mean values of the parameters from preliminary experiment. Four biological replicates in* in vitro* study or ten mice per group in* in vivo* study were required with a type I error of 0.05 and at least a power of 80%. The means of each group were compared using one-way analysis of variance (ANOVA) followed by the Student-Newman-Keuls test when the normality and homogeneity of variance assumptions were satisfied; otherwise, ANOVA followed by Student-Newman-Keuls multiple range test was applied. The significance level was set to *p* < 0.05.

## 3. Results

### 3.1. Blood Gas Analysis

The pH, pCO_2_, pO_2_, SBE, Na^+^, and Cl^−^ values did not significantly differ between the control and SP groups (Table 1). The lactate content was significantly higher in the SP group (27.0 ± 0.6 mmol/L) than in the control group (25.1 ± 0.7) (*p* < 0.05).

### 3.2. Haemoglobin Oxygen Saturation Experiments

**Figure 1:**
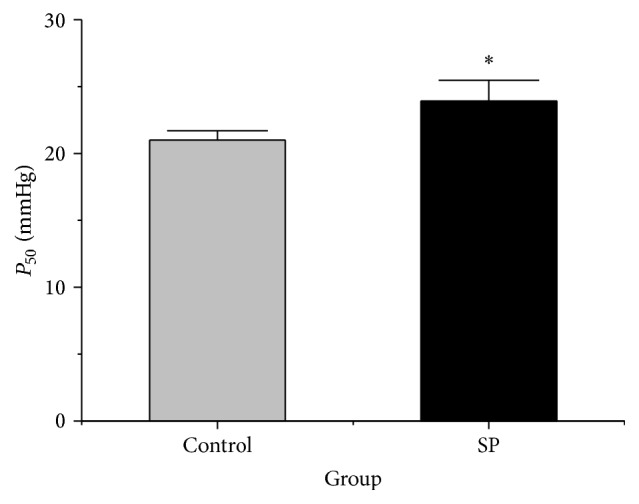
*P*_50_ values in two groups (*n* = 4). The data are plotted as the means ± SD. ^*∗*^ *p* < 0.05 versus the control group.

*P*_50_ value is the pO_2_ at which haemoglobin is half-saturated with oxygen. As shown in Figure 1, the *P*_50_ value in the SP group (23.9 ± 1.5 mmHg) was significantly higher than that in the control group (21.0 ± 0.7 mmHg) (*p* < 0.05).

### 3.3. SOD Activity

**Figure 2:**
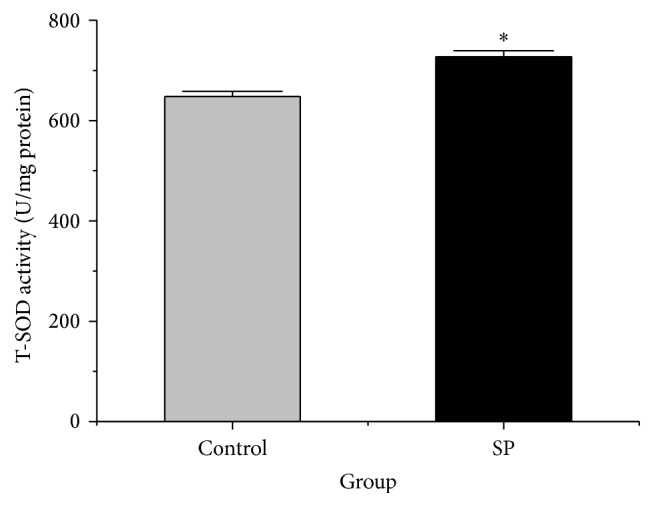
SOD activities of the two groups (*n* = 4). The data are plotted as the means ± SD. ^*∗*^ *p* < 0.05 versus the control group.

SOD is an antioxidant enzyme that has been suggested to be important for ROS elimination. The SOD activity in stored RBCs was significantly higher in the SP group (726.8 ± 12.3 U/mg protein) than in the control group after storage for 14 days (648.1 ± 10.0 U/mg protein) (*p* < 0.05, Figure 2).

### 3.4. Haemolysis and RBC 24 h Recovery

The haemolysis and 24 h recovery of the stored RBCs were measured. The haemolysis of stored RBCs in the SP group (0.77 ± 0.04%) was lower than that in the control group (0.79 ± 0.03%), but this difference was not significant (*p* > 0.05).

The 24 h recovery of stored RBCs was higher in the SP group (77.86 ± 5.4%) than in the control group (75.63 ± 1.8%), but this difference was also not significant (*p* > 0.05).

### 3.5. Plasma Biochemistry

**Figure 3:**
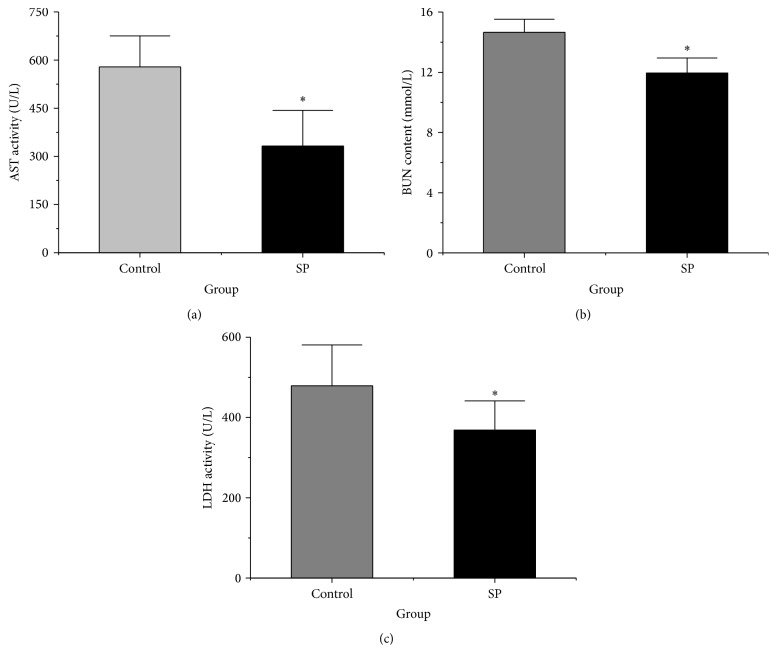
Plasma AST activity, BUN concentration, and LDH activity. (a) Plasma AST activity. (b) Plasma BUN concentration. (c) Plasma LDH activity. The data are plotted as the means ± SD (*n* = 10). ^*∗*^ *p* < 0.05 versus the control group.

The plasma biochemistry was investigated in this study. As shown in Figure 3(a), the activity of AST was significantly lower in the SP group (332 ± 111.2 U/L) than in the control group (579 ± 96.5 U/L) (*p* < 0.05). The BUN concentration was also significantly lower in the SP group (11.9 ± 1.0 mmol/L) than in the control group (14.6 ± 0.8 mmol/L) (*p* < 0.05, Figure 3(b)). The LDH activity was also significantly lower in the SP group (368.8 ± 72.5 U/L) than in the control group (479.0 ± 101.6 U/L) (*p* < 0.05, Figure 3(c)).

### 3.6. Liver Lipid Peroxidation

**Figure 4:**
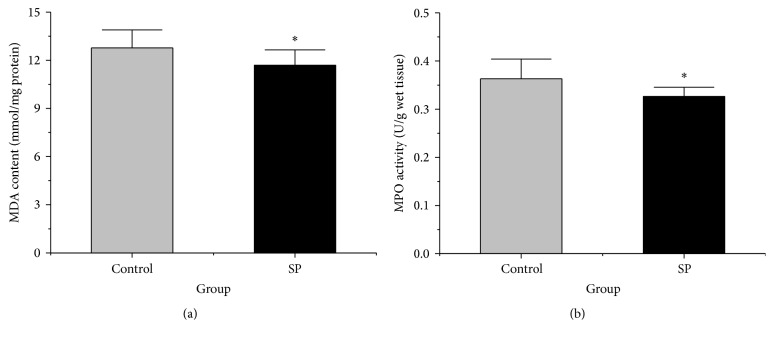
MDA and MPO levels in the livers of the two groups. (a) Liver MDA concentration of the two groups (*n* = 10). (b) Liver MPO activity of the two groups (*n* = 10). The data are plotted as the means ± SD. ^*∗*^ *p* < 0.05 versus the control group.

To assess the tissue lipid peroxidation levels, the MDA concentration in the liver was measured. As shown in Figure 4(a), the concentration of MDA was significantly lower in the SP group (11.69 ± 0.95 mmol/mg protein) than in the control group (12.77 ± 1.11 mmol/mg protein) (*p* < 0.05).

### 3.7. Liver Neutrophil Accumulation

The neutrophil accumulation level in the liver was measured by determining the MPO activity. The MPO activity in the liver was significantly reduced in the SP group compared with the control group (*p* < 0.05, Figure 4(b)).

### 3.8. Hepatic Levels of Inflammatory Cytokines

**Figure 5:**
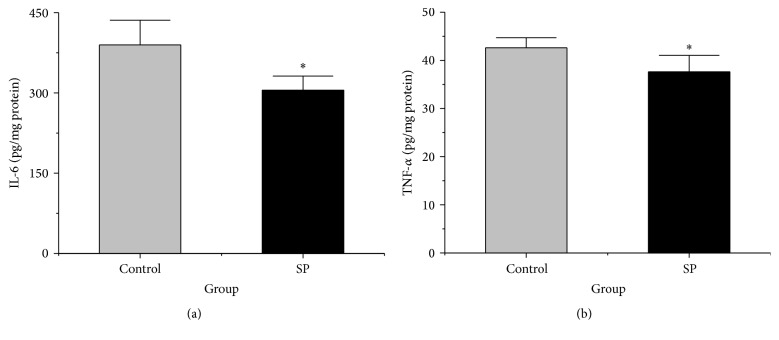
Levels of IL-6 and TNF-*α* in liver of the two groups. (a) Liver IL-6 levels of the two groups (*n* = 10). (b) Liver TNF-*α* levels of the two groups (*n* = 10). The data are plotted as the means ± SD. ^*∗*^ *p* < 0.05 versus the control group.

The increase in IL-6 in the liver was significantly suppressed in the SP group compared with the control group (*p* < 0.05, Figure 5(a)). The level of TNF-*α* in the liver was also lower in the SP group than in the control group (*p* < 0.05, Figure 5(b)). These results suggest that SP can reduce the systemic release of inflammatory cytokines compared with the control group during RBC storage.

### 3.9. Liver Histology

**Figure 6:**
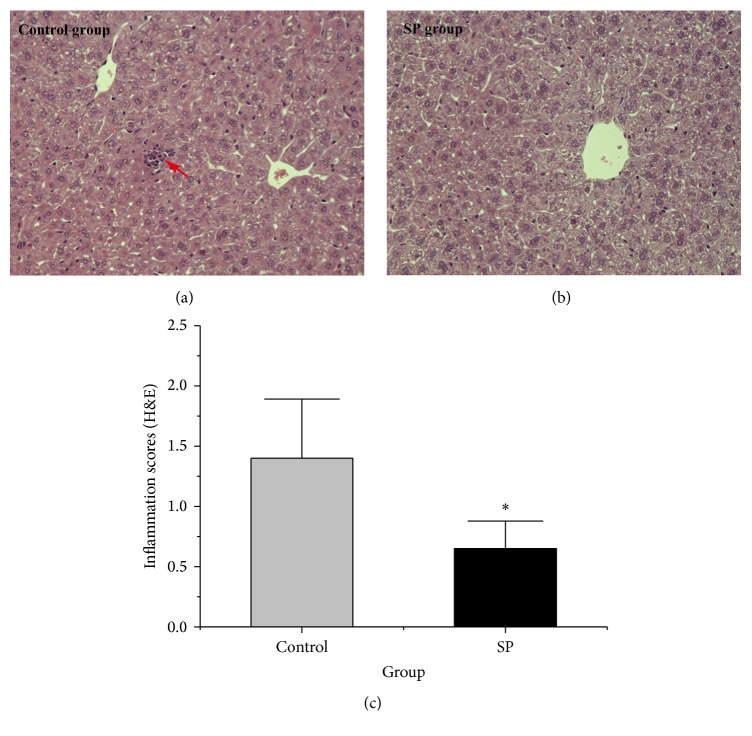
Liver histology. (a and b) Representative haematoxylin-and-eosin-stained images of liver tissues from both groups. (c) Quantification analysis of inflammatory cell infiltration in liver. Data are plotted as the means ± SD. ^*∗*^ *p* < 0.05 versus the control group. The foci of inflammation cell infiltration are indicated by an arrow.

As shown in Figure 6, the livers in the control group exhibited neutrophil infiltration, but these phenomena were less pronounced in the SP livers. The inflammation scores were significantly lower in the SP group than in the control group (*p* < 0.05, Figure 6(c)).

## 4. Discussion

In this study, the effects of adding SP during RBC storage on the storage lesions of RBCs* in vitro* and the organ injury after transfusion were evaluated in a murine model. Our experiments indicated that SP can restore the oxygen-carrying capacity of RBCs during storage and decrease the AST activity, BUN concentration, and LDH activity in the plasma of the recipients 2 h after the transfusion of stored RBCs. The addition of SP during RBC storage also decreased the MDA concentration, the MPO activity, and the IL-6 and TNF-*α* levels in the liver after transfusion compared with the control group. Taken together, our findings suggest that the addition of SP to stored RBCs can attenuate RBC storage lesions and subsequent liver injury in a murine transfusion model.

SP may benefit the oxygen-carrying capacity of RBCs because it not only provides a substrate for energy metabolism but also acts as an antioxidant during RBC storage. Pyruvate is the key intermediate product of the glycometabolism pathway and can be catalysed into lactate, promoting the generation of ATP and 2,3-DPG [35]. A previous study indicated that the ATP and 2,3-DPG levels in RBCs significantly decrease during storage [16]. SP may restore the oxygen-carrying capacity of stored RBCs by improving energy metabolism. In addition, the oxygen-carrying capacity of RBCs is decreased when haemoglobin is oxidized to methemoglobin [36]. SP may restore the oxygen-carrying capacity of RBCs by attenuating the oxidation of haemoglobin during RBC storage.

The method of directly labelling RBCs with FITC has been reported previously [30]. We measured the 24-h survival of fresh RBCs (Table  1S (in Supplementary Material available online at http://dx.doi.org/10.1155/2016/3549207), 97.6 ± 1.2%), and it was similar to the survival reported in a previous study [25]. This result indicated that the FITC-labelling protocol did not negatively affect RBC viability.

The protective effect of sodium pyruvate on RBCs stored in CPDA-1 versus more modern storage solutions (i.e., SAGM, AS-1) still needs to be evaluated. In this study, we reported that the addition of sodium pyruvate to RBCs during storage in CPDA-1 has a beneficial effect on storage lesions* in vitro* and subsequently alleviates liver injury following transfusion of the stored RBCs* in vivo*. This beneficial effect may be due to an improvement in energy metabolism, oxidative state, intracellular acidosis, or the oxygen-carrying capacity of the RBCs [20, 37]. Both SAGM and AS-1 solutions contain mannitol and sodium chloride, which can maintain the integrity of the cell membrane, while the CPDA-1 solution does not. However, SAGM and AS-1 storage solutions do not contain ingredients that specifically target reactive oxygen species, intracellular acidosis, or energy metabolism. Thus, we believe that the addition of sodium pyruvate to the modern storage solutions can still provide additional protective effects to the RBCs.

Addition of sodium pyruvate to stored RBCs has been investigated previously for the purpose of improving the quality of stored human RBCs. A study by Beutler et al. has indicated that pyruvate can maintain ATP levels during human RBCs storage at concentrations ranging from 0.3 to 3 mM, whereas 2,3-DPG cannot [23, 38]. Dawson et al. reported that higher pyruvate concentrations (40–320 mM) could maintain elevated or normal 2,3-DPG levels, but decreased ATP levels in stored human RBCs [24]. In our study, the sodium pyruvate dose (2.5 mM) was selected based on previous studies and our preliminary experiment. The reported therapeutic* in vitro* dose for sodium pyruvate in previous studies has mostly been between 1 and 4 mM [23, 39, 40]. In our preliminary experiment, we observed that 2.5 mM dose was superior for *P*_50_ values* in vitro*, compared with a dose of 1 mM, 5 mM, or 10 mM. We found the pyruvate (2.5 mM) could improve the ATP levels [41] and oxygen-carrying capacity of stored murine RBCs. More studies are required to optimize the pyruvate concentration for improving the quality of stored RBCs. In addition, previous studies have indicated that sodium pyruvate can attenuate tissue damage in haemorrhagic shock, brain damage, and ischemia-reperfusion injury models. Therefore, the washing procedure prior to transfusion in this study was necessary to exclude the impact of residual sodium pyruvate on liver injury.

A previous study indicated that prolonged RBC storage prior to transfusion is associated with increased rates of infection, multiorgan failure, and mortality in some hospitalized patients [11, 42–44]. Although the precise mechanisms responsible for the deleterious effects of stored RBC transfusion are not fully understood, the reduced deformability of stored RBCs has been considered an important reason for the negative effects observed after the transfusion of stored RBCs [45]. In this study, we found that the addition of SP to stored RBCs can attenuate liver injury after transfusion. The improvement of the membrane deformability of RBCs during storage may contribute to the beneficial effects of SP. SP may improve the membrane deformability of RBCs and maintain the integrity of the RBC membrane by reducing lipid peroxidation. Additional studies should clarify the precise mechanisms through which SP protects organs in the recipient after the transfusion of stored RBCs.

Although only liver injury was evaluated in this study, the addition of SP to stored RBCs may also mitigate nonhepatic organ injury. Our results indicate that the addition of SP to stored RBCs can decrease the plasma BUN concentration (Figure  1S), a conventional marker of kidney injury, after transfusion. In addition, LDH is an important isoenzyme in glycolysis and ubiquitous in the lung, heart, liver, and other tissues. Tissue injury causes the release of LDH into the plasma, which increases the LDH activity in plasma [46]. In this study, the addition of SP to stored RBCs can also inhibit the LDH activity in plasma after transfusion. This result indicates that the addition of SP to stored RBCs may attenuate tissue injury in the recipient after transfusion.

The addition of SP to saline alters the osmolality of the final solution. Previous studies reported that the osmotic pressure can affect the quality of stored RBCs [47, 48]. Increases in osmolality may reportedly promote the leakage of potassium, disturbing the ionic equilibrium, which consequently decreases the quality of stored RBCs [49]. In this experiment, the addition of SP to saline slightly elevated the osmolality of the final solution (SP group: 376 ± 2 mOsm/kg; control group: 368 ± 1 mOsm/kg), but SP was found to benefit stored RBCs. These results indicate that changes in the osmolality due to the addition of SP during RBC storage may not contribute to the beneficial effects of SP observed in this study.

In this study, the murine system is a tractable platform in which to rapidly study the adverse consequences of transfusion of stored RBCs* in vivo* and the improvement of adding SP during RBC storage. However, in view of the difference between mouse model and human in RBC storage, these observations from mouse model may not always be directly applicable to humans [50–52]. Thus subsequent studies are still needed to test these hypotheses in human RBC storage systems.

## 5. Conclusions

In conclusion, the addition of SP solution to RBCs during storage restores the oxygen-carrying capacity of RBCs and subsequently alleviates liver injury after the transfusion of stored RBCs. Although these findings warrant further study, our results indicate that the addition of SP to stored RBCs may be a promising strategy for avoiding the adverse effects of transfusing stored RBCs. Subsequent focused studies in humans will be required to test these hypotheses in human RBC storage systems.

## Supplementary Material

Supplementary Table 1. Blood gas and 24-hour recovery of the fresh RBCs.
Supplementary Figure 1: Plasma AST activity, BUN concentration and LDH activity.

## Figures and Tables

**Table 1:** Blood gas of the RBCs after 14 days of storage (*n* = 4).

Index	Control group	SP group
pO_2_ (mmHg)	77.5 ± 3.9	78.4 ± 2.4
pCO_2_ (mmHg)	134 ± 5.3	140 ± 5.1
sO_2_ (%)	48.6 ± 2.0	44.7 ± 1.8
CHb (g/dL)	24.12 ± 1.3	23.8 ± 1.2
Hct (%)	73.8 ± 3.9	72.7 ± 2.6
Na^+^ (mmol/L)	91.5 ± 2.0	92.1 ± 1.6
Cl^−^ (mmol/L)	78.3 ± 2.8	79.3 ± 1.4
Lactate (mmol/L)	25.1 ± 0.7	27.0 ± 0.6^*∗*^

^*∗*^ *p* < 0.05 versus the control group.

## Abbreviations

AST: Aspartate aminotransferase
BUN: Blood urea nitrogen
CPDA-1: Citrate-phosphate-dextrose-adenine-1
FITC: Fluorescein isothiocyanate
FDA: Food and Drug Administration
IL-6: Interleukin-6
MDA: Malondialdehyde
MPO: Myeloperoxidase
LDH: Lactate dehydrogenase
pCO_2_: Partial pressure of carbon dioxide
pO_2_: Partial pressure of oxygen
RBCs: Red blood cells
ROS: Reactive oxygen species
SOD: Superoxide dismutase
SP: Sodium pyruvate
TCA: Tricarboxylic acid
TNF-*α*: Tumor necrosis factor-alpha.
