# Supplementary material for: Addition of Sodium Pyruvate to Stored Red Blood Cells Attenuates Liver Injury in a Murine Transfusion Model
Source: Mediators Inflamm. 2016 Sep 26;2016:3549207. doi: 10.1155/2016/3549207 (PMC5056311; doi:10.1155/2016/3549207)

**Supplementary table 1. Blood gas and 24-hour recovery of the fresh RBCs**

| Index | Fresh RBCs group |
| --- | --- |
| pO_2_（mmHg） | 148.4±3.74 |
| pCO_2_（mmHg） | 42.0±5.12 |
| sO_2_（%） | 87.9±0.12 |
| _C_Hb（g/dL） | 22.8±0.82 |
| Hct（%） | 72.6±1.66 |
| Na^＋^（mmol/L） | 160.8±2.36 |
| Cl^－^（mmol/L） | 72.3±2.06 |
| The 24-hour recovery | 97.6±1.2% |
| P_50_ (mmHg) | 41.68±1 |

**Supplementary figure 1: Plasma AST activity, BUN concentration and LDH activity. (A) Plasma AST activity. (B) Plasma BUN concentration. (C) Plasma LDH activity. The data are plotted as the means ± SD (n=10).** # *p*<0.05 versus the blank group, & *p*<0.05 versus the Fresh RBCs group, **p*<0.05 versus the control group.


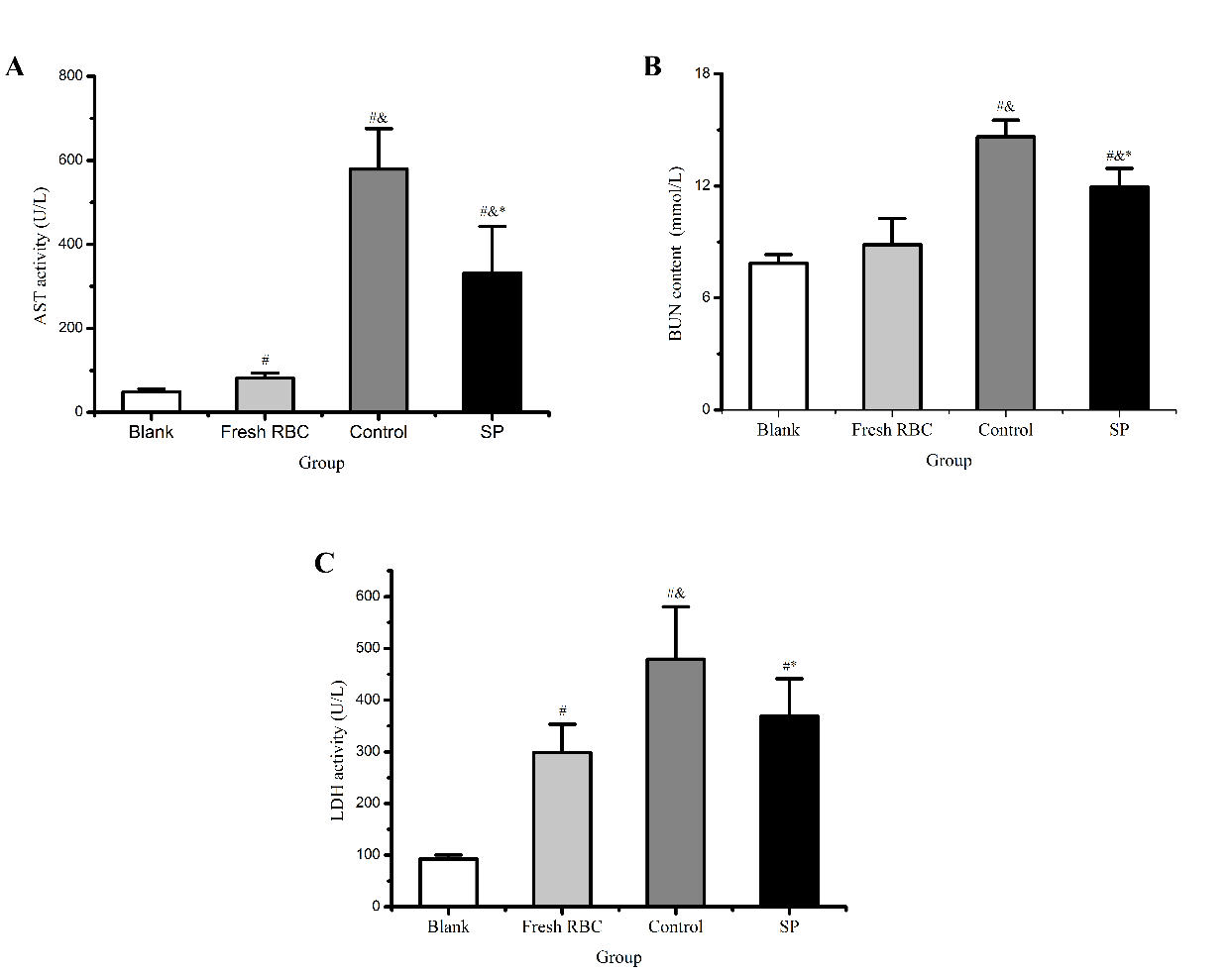

Supplement: Supplementary file 1 — Supplementary Table 1. Blood gas and 24-hour recovery of the fresh RBCs. Supplementary Figure 1: Plasma AST activity, BUN concentration and LDH activity. [file 3549207.f1.docx]
